# Oseltamivir resistance in an influenza A (H3N2) virus isolated from an immunocompromised patient during the 2014–2015 influenza season in Alberta, Canada

**DOI:** 10.1111/irv.12415

**Published:** 2016-08-08

**Authors:** Ahsan Chaudhry, Nathalie Bastien, Yan Li, Allison Scott, Kanti Pabbaraju, Douglas Stewart, Sallene Wong, Steven J. Drews

**Affiliations:** ^1^ Departments of Oncology and MedicineUniversity of CalgaryCalgaryABCanada; ^2^ National Microbiology LaboratoryWinnipegMBCanada; ^3^ Surveillance and Assessment, Accountability and PerformanceEdmontonABCanada; ^4^ ProvLab Alberta (Calgary)CalgaryABCanada; ^5^ ProvLab Alberta (Edmonton)EdmontonABCanada; ^6^ Department of Laboratory Medicine and PathologyUniversity of AlbertaEdmontonABCanada

**Keywords:** H3N2, influenza, neuraminidase inhibitors, reduced susceptibility, resistance, sequence analysis

## Abstract

This manuscript describes the identification of an oseltamivir‐resistant influenza A (H3N2) virus in a respiratory specimen collected from an immunocompromised patient in Alberta, Canada, during the 2014–2015 influenza season. Following treatment with oseltamivir, neuraminidase (NA) gene sequencing indicated the presence of an R292K mutation. Phenotypic susceptibility testing by the NA‐Star assay indicated a highly reduced inhibition by oseltamivir and normal inhibition by zanamivir. The use of zanamivir following identification of the oseltamivir‐resistant strain, combined with a partial immune reconstitution, was followed by a suggested decrease in the nasopharyngeal viral load in the nasopharynx and clinical improvement of the patient.

## 1 Introduction

Within influenza A (H3N2) viruses, a variety of amino acid mutations in the neuraminidase (NA) gene (e.g., R292K, E119V, I122V, N294S, Δ244‐247, and Q136K) have been detected in viruses from respiratory clinical samples and may cause reduced inhibition to susceptibility in vitro.^1^, ^2^ However, the clinical impact of these mutations is still not clear, and the World Health Organization Antiviral Working Group has suggested full NA sequencing should be done for screening of known or new mutations. Ideally, if viruses can be isolated in cell culture, a NA phenotypic assay (using a fluorometric or luminescent substrate) should be performed.^1^ With the recent dominance of influenza A (H3N2) viruses, there have been several descriptions of A (H3N2) viruses carrying mutation in the NA, especially in immunocompromised patients.^2^, ^3^ This short report describes the identification of an R292K mutation in the NA of a seasonal influenza A (H3N2) virus from an immunocompromised patient. This was the only identified oseltamivir‐resistant influenza A (H3N2) viral isolate characterized in Canada during the 2014–2015 influenza season.

## 2 Methods

**Figure 1:**
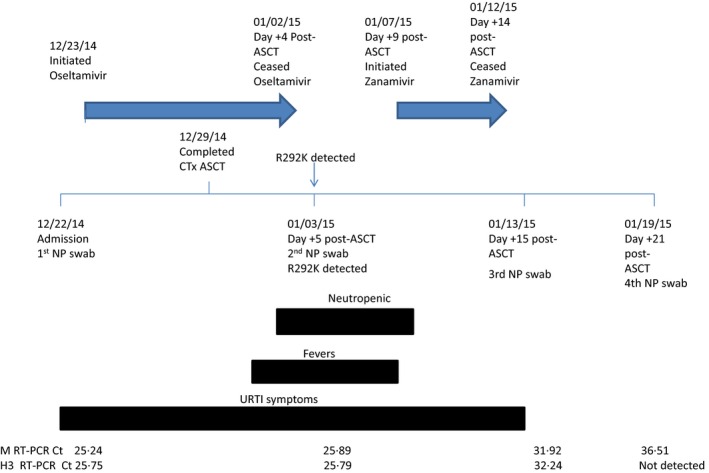
Laboratory test results and clinical progression of an immunocompromised patient infected with an oseltamivir‐resistant influenza A (H3N2) virus. Arrows indicate periods of antiviral administration in relation to timeline. Solid blocks indicate key clinical presentations in relation to timeline. *C*_t_ values for the influenza A virus M and H3 genes are listed at the bottom of the figure in relation to timelines and represent the four specimens collected. Hemagglutinin (HA) sequencing allowed the specimens to be subgrouped as a 3C.2a virus for three specimens, the fourth specimen having a viral load too weak to allow for HA sequencing. Neuraminidase sequencing indicated that the first NP swab collected contained a wild‐type virus with R292 in N2. The second specimen collected contained a R292K mutation in N2 and had a highly reduced inhibition by oseltamivir (20884.78‐fold above NI) but a normal inhibition by zanamivir (3.21‐fold above NI). A decrease in viral load, as evidenced by increasing *C*_t_ values for M and H3 RT‐PCR, was noted for the third and fourth specimens following treatment with zanamivir, an improved immune status of the patient, and resolution of fevers and upper respiratory tract infection (URTI) symptoms. Abbreviations are as follows: autologous stem cell transplantation (ASCT), chemotherapy (CTx), cycle threshold (*C*_t_), nasopharyngeal (NP), and reverse transcriptase polymerase chain reactions (RT‐PCRs).

Nasopharyngeal (NP) specimens were taken at four time points (Fig. 1). Pre‐admission swabs from the start of symptoms were not available. Real‐time reverse transcription PCRs were undertaken for influenza A matrix (M) gene detection and subtyping of H3 and pdm09 H1 viral targets. These were as per routine laboratory standard operational procedures on all NP specimens. Sequencing of the NA gene was undertaken as previously described.^4^ Careful manual examination of the electropherogram at bases that code for amino acid 119–294 was performed to monitor for the presence of previously described single nucleotide polymorphisms (mixed or in total), described above as well as deletions.

For strain analysis from the primary specimen, hemagglutinin (HA) sequencing and alignment were done as previously described.^4^, ^5^, ^6^

The NA‐Star assay was performed according to manufacturer's recommendations. Two real‐time RT‐PCR‐positive specimens were cultured on MDCK and MDCK‐SIAT1 cells using previously published protocols at the National Microbiology Laboratory, Winnipeg, Canada.^7^, ^8^ Reduced inhibitor susceptibility was expressed as fold changes in IC_50_ compared to wild‐type (NAI sensitive) virus (influenza A/Switzerland/9715293/2013‐like).^2^ Reduced inhibitor susceptibility was then classified by the World Health Organization (WHO) Antiviral Working Group (AVWG) Proposed Susceptibility Criteria for Surveillance and Reporting.^1^ Briefly, normal inhibition (NI) is defined as <10‐fold above NI. Reduced inhibition (RI) is defined as 10‐ to 100‐fold above NI, while highly reduced inhibition (HRI) is >100‐fold above NI.

Routine hematology analysis was performed on the patient between December 23, 2015 and January 19, 2015.

Clinical information was provided by the managing physicians. As this was a case report, and was undertaken as part of normal surveillance activities and public health activities for the emergence of antiviral resistant strains of influenza, research ethics board clearance was not required by the University of Alberta, Research Ethics Office. However, since the manuscript involved a detailed rereview of the patient record, a representative of the data custodian, Alberta Health, was engaged in the analysis.

## 3 Results

The patient was a 51‐year‐old male previously diagnosed with a stage IIA bulky diffuse large B‐cell lymphoma in April 2011. On November 4, 2014, a relapse in lymphoma involving liver and bulky retroperitoneal nodes, associated with hypercalcemia and incidental positive hepatitis C serology on routine pre‐transplant investigation, was diagnosed. Salvage treatment was administered in the hospital starting November 12, 2014 using one cycle of R‐DICEP (rituximab and dose‐intensive cyclophosphamide, etoposide, cisplatin).

On December 22, 2014, the patient was admitted to the hospital to begin R‐BEAM conditioning (rituximab, carmustine, etoposide, cytarabine, melphalan) prior to autologous stem cell transplantation (ASCT). On admission, the patient had symptoms of a viral upper respiratory tract infection. An NP swab was performed, which documented influenza A (H3N2) virus infection. The patient was then treated with oseltamivir 75 mg orally twice daily for 10 days while he completed R‐BEAM and ASCT on December 29, 2014 (Fig. 1).

Lymphocyte counts for the whole period (December 23, 2014–January 19, 2014) were below normal (0.7–3.5 10^9^/L) and ranged from a starting level of 0.2 × 10^9^/L to lows of 0 × 10^9^/L with a level of 0.4 10^9^/L on January 19, 2015. Lymphocyte reconstitution was not evident during this time period. Total white blood cell (WBC) counts were below normal (<4.0 × 10^9^/L) from Day +2 to Day +9 post‐ASCT with a range of 0.1–3.0 × 10^9^/L. Total WBC counts were normal (4.0–11.0 × 10^9^/L) or slightly elevated (>11.0 × 10^9^/L) from days +10 to +21 post‐ASCT with ranges of 4.0–11.8 × 10^9^/L during this period.

The patient was neutropenic (Day +4 to Day +10) and experienced fevers between Day +2 and Day +9 post‐ASCT. Following treatment with empiric broad spectrum antibacterials, and a negative computerized tomography scan of the chest/abdomen/pelvis, the central line was removed. The patient was neutropenic from Day +3 until Day +10 post‐ASCT (<2.0 × 10^9^/L neutrophils; range 0–1.4 × 10^9^/L neutrophils). Following that period, from Day +10 to Day +21 post‐ASCT, the neutrophils normalized (2.0–8.0 ×10^9^/L) or were slightly elevated (>8.0 × 10^9^/L) with counts ranging from 2.0 to 8.2 × 10^9^/L neutrophils. On Day +5 post‐ASCT, the patient was still symptomatic and febrile and his NP swab continued to be positive for influenza A virus. Zanamivir was initiated on Day +9 post‐ASCT at a dose of 10 mg inhalation twice daily for 5 days (Fig. 1). Upper respiratory tract infection (URI) symptoms and fever resolved by Day +15 post‐ASCT; NP swabs continued to be positive for influenza A virus on days +15 and +21 post‐ASCT. No further NP swabs were performed because the patient remained clinically well.

The cycle threshold (*C*_t_) of real‐time RT‐PCR for the M and H3 genes of influenza A viruses for all specimens collected is listed at the bottom of Fig. 1. The first NP specimen was collected at admission (December 22, 2014), with a strong positive influenza A virus M gene signal. Additional NP specimens were collected over the period January 03, 2015–January 19, 2015 with positive influenza A virus test results and increasing (*C*_t_) values for the matrix and H3 genes which can be interpreted as decreasing viral load.

Sequence analysis of the NA gene from the primary specimen collected on January 3, 2015 indicated the presence of a single R292K mutation. In contrast, sequence analysis of the primary specimen collected on admission did not have this mutation and was R292 wild type. We did not note mixed subpopulations in the analysis. No other mutations were evident in the NA gene of either specimen and we could not detect any mixed subpopulations at position 292 by visual inspection of the electropherogram. Sequencing of primary specimens from Day +15 and Day +21 post‐ASCT was not possible due to low viral loads.

The sequence analysis of the HA gene from clinical specimens collected at admission, Day +5, and Day +15 post‐ASCT indicated that the viruses are related to A/Hong Kong/4801/2014 (H3N2) reference strain and belong to subgroup 3C.2a. Sequencing of HA from the Day +21 post‐ASCT specimen was not undertaken due to low viral load.

The drug‐sensitive control strain (A/Switzerland/9715293/2013‐like [H3N2]) had IC_50_ values of 0.46 nmol/L for oseltamivir and 1.31 nmol/L for zanamivir. The IC_50_ values for the isolate from the specimen collected on admission were 0.68 nmol/L for oseltamivir and 1.49 nmol/L for zanamivir. The IC_50_ values from the Day +5 post‐ASCT isolate were elevated compared with the wild‐type control with an IC_50_ of 9607 nmol/L for oseltamivir and 4.20 nmol/L for zanamivir. Using the proposed AVWG criteria, the isolate collected on admission had normal inhibition by oseltamivir (1.48‐fold above NI) and zanamivir (1.38‐fold above NI). In contrast, the clinical isolate collected at +Day 5 had a HRI by oseltamivir (20884.78‐fold above NI) and normal inhibition by zanamivir (3.21‐fold above NI).

## 4 Discussion

This study describes the identification of an oseltamivir‐resistant influenza A (H3N2) virus specimen during the 2014–2015 influenza season in Canada. The R292K mutation has been associated with in vitro reduced susceptibility to oseltamivir, zanamivir, and peramivir, with larger IC_50_ values against oseltamivir (usually >10 000 nmol/L) and several logs lower IC_50_ values for zanamivir (3–20 nmol/L) and peramivir (14 n nmol/L). Oseltamivir resistance is relatively infrequent but has been described as a transient phenomenon in immunocompromised patients.^3^ Thus, publication of these rare cases is important for the better understanding of these mutations and their association with phenotypic resistance and clinical outcomes.

In this case, the patient excreted influenza A (H3N2) virus for an extended period of time. Interestingly, the viral load based on *C*_t_ values for the influenza A virus M gene did not decrease during the time period when the patient was treated by oseltamivir. A persistent high viral load (suggested by low M gene *C*_t_ values) during antiviral treatment may alert clinicians and virologists on the possible development of antiviral resistance. The phenotypic susceptibility data for the isolate from Day +5 post‐ASCT indicate HRI to oseltamivir, suggesting that this agent would not be effective against the mutant strain. However, we cannot speculate on how long the patient would have been treated with a potentially decreasingly effective antiviral agent. We do know from the case description that this R292K mutant was identified during a time range when the patient could be considered highly immunocompromised, with neutropenia.^9^ Immunocompromised patients can shed influenza virus for extended periods of time and this may promote the development of resistant or non‐susceptible strains as homogenous or heterogeneous populations over time.^2^, ^3^ The R292K mutation has been associated with decreased viral fitness in ferret models and it is possible that reconstitution of the patient's immune system would have at least partly cleared this strain.^10^

The phenotypic susceptibility test data also suggest that the isolate from Day +5 post‐ASCT would have had normal levels of inhibition if treated with zanamivir. From Fig. 1, zanamivir was introduced during a period of time when the patient was still strongly immunosuppressed. Following treatment, both Day 15+ post‐ASCT and Day 21+ post‐ASCT specimens had a noticeable increase in *C*_t_ values of the influenza A virus M gene and HA RT‐PCRs, suggesting a decrease in viral load. Since susceptibility analysis was not possible on these later specimens, it is unknown whether mutations other than R292K would have arisen and whether these would have been associated with changes in zanamivir susceptibility.^2^ Thus, the noted clinical recovery of this patient may have been due to a variety of factors, including improvements in the immune status as evidenced by normalization of both neutrophil and WBC levels (Day + 10 post‐ASCT), a potential low‐fitness strain, and/or appropriate use of an effective antiviral agent. However, it should be noted lymphocyte levels were below normal for the period described in this case study.

Genotyping of influenza strains has not always provided definitive evidence of reduced sensitivity to NA inhibitors in influenza A (H3N2) virus. The gold standard is still phenotypic resistance testing using a variety of well‐published techniques.^11^ However, questions still arise regarding the mutations generated in cell culture systems^12^ which may lead to artifactual results.^13^ There are also some concerns that the NA‐Star assay, a chemiluminescent assay, may not be able to detect low‐level increases in IC_50_ within isolates containing the R292K polymorphism that were detectable when fluorescent assays were used.^14^ The continued description and comparison of influenza A (H3N2) cases with resistance to NA inhibitors will lead to a better understanding of antiviral susceptibility, and inferences can be made from primary sequence analysis of specimens with less emphasis of phenotypic analysis from culture isolates.
